# Positive Allosteric Modulation of CB1 and CB2 Cannabinoid Receptors Enhances the Neuroprotective Activity of a Dual CB1R/CB2R Orthosteric Agonist

**DOI:** 10.3390/life10120333

**Published:** 2020-12-08

**Authors:** Beatrice Polini, Chiara Cervetto, Sara Carpi, Simone Pelassa, Francesca Gado, Rebecca Ferrisi, Simone Bertini, Paola Nieri, Manuela Marcoli, Clementina Manera

**Affiliations:** 1 Department of Pharmacy, University of Pisa, 56126 Pisa, Italy; beatrice.polini@farm.unipi.it (B.P.); sara.carpi@unipi.it (S.C.); francesca.gado@for.unipi.it (F.G.); rebeccaferrisi@hotmail.it (R.F.); simone.bertini@unipi.it (S.B.); paola.nieri@unipi.it (P.N.); 2 Department of Pharmacy, Section of Pharmacology and Toxicology, University of Genova, 16126 Genova, Italy; cervetto@difar.unige.it (C.C.); pelassa@difar.unige.it (S.P.); marcoli@difar.unige.it (M.M.)

**Keywords:** endocannabinoids system, cannabinoid receptor type 2, cannabinoid receptor type 1, positive allosteric modulator, microglial cell, glutamate release

## Abstract

Preclinical studies highlighted that compounds targeting cannabinoid receptors could be useful for developing novel therapies against neurodegenerative disorders. However, the chronic use of orthosteric agonists alone has several disadvantages, limiting their usefulness as clinically relevant drugs. Positive allosteric modulators might represent a promising approach to achieve the potential therapeutic benefits of orthosteric agonists of cannabinoid receptors through increasing their activity and limiting their adverse effects. The aim of the present study was to show the effects of positive allosteric ligands of cannabinoid receptors on the activity of a potent dual orthosteric agonist for neuroinflammation and excitotoxic damage by excessive glutamate release. The results indicate that the combination of an orthosteric agonist with positive allosteric modulators could represent a promising therapeutic approach to the treatment of neurodegenerative disorders.

## 1. Introduction

During the last 50 years, with the extension of lifespan, the incidence of neurodegenerative disorders has been increasing and is becoming an important public health burden. Despite years of intense research, current treatments do not allow effective control of these disorders, and thus, new therapeutic strategies are needed.

In neurodegenerative disorders, damage to neurons and glial cells is produced by various cytotoxic events acting together, such as excitotoxicity, oxidative stress and neuroinflammation, that interfere with cell homeostasis and integrity. Therefore, it appears reasonable that a new therapeutic strategy could be represented by the combination of different therapeutic agents able to control excitotoxicity and neuroinflammation simultaneously.

In this context, targeting the endocannabinoid system (ECS) could present great advantages. The ECS is a complex modulatory system that includes endogenous bioactive lipids known as endocannabinoids (ECs), of which the two best known and characterized are anandamide (AEA) and 2-arachidonoylglycerol (2-AG); their synthesizing and degrading enzymes, such as fatty acid amide hydrolase (FAAH) and monoacylglycerol lipase (MAGL); the endocannabinoid membrane transporter (EMT); and at least two G-protein coupled receptors, cannabinoid receptors type-1 (CB1R) and type-2 (CB2R) [1]. CB1R is mostly present in the central nervous system (CNS) but also in non-neuronal tissues; in particular, its presence has been detected at the central and peripheral nerve terminals where it mediates the inhibition of transmitter release. Regarding CB2R, its expression was identified in a few areas of the CNS (including microglia) and it is predominant in immune cells in which CB2R is important for the modulation of interleukin release and cell migration [2,3]. The ECS is involved in the regulation of cell, tissue and organ homeostasis, neurotransmitter release, synaptic plasticity, brain development and cytokine release from microglia; and therefore, the deregulation of ECS is implicated in multiple neurological disorders [4].

Numerous preclinical studies highlighted that compounds targeting the ECS could be useful for developing novel therapies against neurodegenerative disorders based on several of their features: the locations of the endocannabinoid targets in cell substrates are critical for neuronal survival; the ability to preserve, rescue or replace neurons and glial cells against cytotoxic insults, and therefore the broad-spectrum neuroprotective profile.

In detail, the multiplicity of action of cannabinoids permits the reduction of the excitotoxicity by acting through neuronal CB1R, limiting glutamate release [5]. Excessive release of glutamate generates accumulations of toxic concentrations of intracellular calcium, resulting in excitotoxicity, a process common to many brain disorders that often leads to neuronal death [6].

Furthermore, the activation of CB2R decreases microglia-derived neuroinflammation, modifying the ratio between pro- and anti-inflammatory cytokines released by these cells [7,8,9,10]. CB2R was reported to be increased 10-fold in inflamed microglial cells, but this up-regulation is not accompanied by an increased production of the CB2R endogenous ligand 2-AG [11]. These pieces of evidence indicate the potential of CB2R as a therapeutic target for the management of neuro-inflammation.

In spite of their theoretical usefulness, the use of CB1R and CB2R orthosteric agonists chronically has several disadvantages, limiting their usefulness as clinically relevant drugs. In particular, CB1R orthosteric agonists induce psychotropic effects, strong mood alterations, acute psychosis and cognitive and motor impairments [12]. On the contrary, CB2R ligands, which do not produce psychotropic effects, appear to be promising drugs for treating several neuro-inflammatory diseases. However, only a few synthetic CB2R agonists have reached an advanced stage of clinical trials (from ClinicalTrials.gov: GW842166X, CP-55,940, S-777469 and JTE-907), probably because of the predominance of CB2R on immune cells, whose activation might cause immunosuppression [13].

For the above-mentioned limitations, alternative medicinal chemistry approaches are now being studied to develop more efficacious and safer CBR ligands. Positive allosteric modulators (PAMs) might represent a promising approach to achieve the potential therapeutic benefits of orthosteric CBR agonists by limiting their adverse effects [14].

Allosteric modulators interact with topographically different sites from the orthosteric ligand-binding pocket, and by modifying the conformations of their respective receptor proteins, they modulate the affinity and/or efficacy of orthosteric ligands without being able to activate the receptor on their own. PAMs allow a pharmacological fine-tuning of receptor activation, enhancing the activity and at the same time maintaining spatial and temporal control. This permits an improved safety profile with respect to the direct orthosteric activation alone [15,16,17,18].

**Figure 1:**
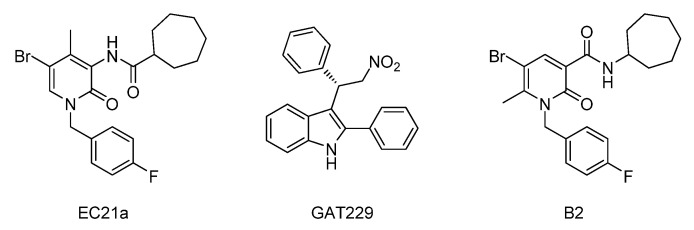
Structures of CBR PAMs, EC21a and GAT229, and of dual orthosteric CBR agonist B2.

Recently, GAT229 and EC21a (Figure 1) have been reported to modulate CB1R and CB2R respectively, through the allosteric site of each receptor [19,20]. EC21a was recently reported by us as the first synthetic CB2R PAM displaying antinociceptive activity in an in vivo experimental mouse model of neuropathic pain [20]. Its allosteric behavior was determined through [^3^H]CP55940 and [^35^S]GTPγS binding assays. In particular, EC21a was found able to enhance the ability of CP55940 and that of 2-AG, but not that of AEA, to stimulate [^35^S]GTPγS binding to CB2Rs; it also showed probe-dependent activity [20]. GAT229 is the S enantiomer of the 2-phenylindole derivative GAT211. This last compound was the first well-characterized CB1R ago-PAM; its R-(+)-enantiomer, GAT228, was displayed as a partial allosteric agonist with weak PAM activity, and the S-(−)-enantiomer GAT229 was a pure CB1R PAM [19].

Derivative B2 (Figure 1) was synthesized by us, and it belongs to a series of 2-oxo-pyridin-3-carboxamide derivatives that have shown high affinity in the nanomolar range and agonist activity on both CBRs [21]. It behaved as a dual orthosteric agonist at CB1R and CB2R. In particular, B2 showed activity against neuroinflammation and excitotoxic damage by excessive glutamate release in in vitro assays [21]. Indeed, it could reduce the release of glutamate from rat glutamatergic nerve terminals through interaction with CB1R, while modulating the production of the pro-inflammatory interleukins IL-1β and IL-6 and of the anti-inflammatory IL-10 via CB2R activation [21]. Notably, the compound exhibited in vivo antinociceptive effects in a neuropathic pain experimental model and was effective in an experimental model of multiple sclerosis [21].

The goal of the present study was to determine whether the positive allosteric ligands GAT229 and EC21a of CB1R and CB2R respectively, can modulate the activity of the potent dual CB1R/CB2R orthosteric agonist B2 regarding neuroinflammation by controlling the release of pro- and anti-inflammatory cytokines and regarding the neurotoxicity by reducing the release of glutamate.

## 2. Results

### 2.1. EC21a Potentiated the CB2R-Mediated Effects on IL Release of B2

To evaluate the ability of EC-21a to act as PAM of CB2R, we evaluated whether it was able to potentiate the activity of the orthosteric agonist B2 on CB2R. With this purpose, we stimulated the BV2 cell line with LPS to induce a pro-inflammatory response that mimicked the status of microglial inflammation. The activated BV2 cells were treated with B2 and EC-21a either alone or in combination. Furthermore, a simultaneous treatment was also performed in the presence of the CB2R antagonist SR144528. As a marker of inflammatory response, we analyzed the release of extracellular interleukins in cell media of stimulated BV-2 cells in all tested conditions.

**Figure 2:**
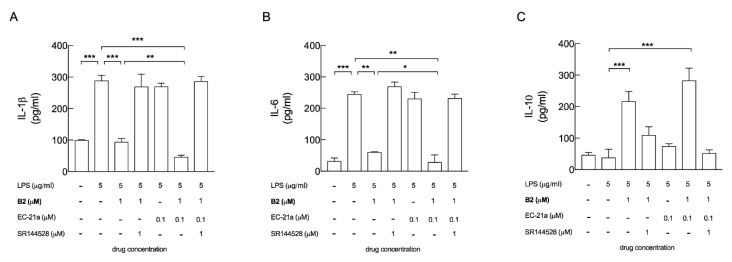
EC-21a potentiated the ability of the CB agonist B2 to decrease the inflammatory phenotype of LPS-stimulated BV2 microglial cells, by the modulation of CB2R. The treatment with EC-21a in the presence of the B2 agonist significantly decreased the release of pro-inflammatory IL-1β (**A**) and IL-6 (**B**) and further increased the release of the anti-inflammatory IL-10 (**C**), compared to the treatment with the B2 agonist alone. The CB2R antagonist SR144528 totally counteracted the anti-inflammatory effects induced by the B2 agonist alone and by the co-treatment (**A**–**C**). Bars represent the release (pg/mL) of ILs in the presence of the drugs at the indicated concentrations. Data represent means ± SD (bars) from three independent experiments, each performed in duplicate. * *p* < 0.05, ** *p* < 0.01 and *** *p* < 0.001—ordinary one-way ANOVA followed by Tukey’s multiple comparison test.

In the in vitro microglial model, LPS stimulus induced a significant increase of the pro-inflammatory IL-1β and IL-6 release, and it did not affect the release of the anti-inflammatory IL-10, compared to unstimulated cells used as the negative control (Figure 2A–C). As expected, the treatment with the CB2R agonist B2 (1 μM) counteracted the inflammatory response induced by LPS—decreasing the IL-1β and IL-6 release (3.1 and 4-fold change, respectively) and increasing the IL-10 release (5.7-fold change) compared to LPS-activated BV2 cells. This modulation of IL release induced by B2 compound was totally reverted in the presence of the CB2R antagonist SR144528 (1 μM).

The treatment with EC21a alone (0.1 μM) did not show any significant effect on the release of all the tested ILs, whose levels appeared comparable to those observed from untreated LPS-stimulated BV2 cells (Figure 2A–C).

On the other hand, 0.1 μM EC21a compound significantly potentiated the ability of the B2 orthosteric agonist (1 μM) to modulate IL release. In detail, the release of the pro-inflammatory IL-1β and the release of IL-6 were significantly decreased (2 and 2.1-fold change), and the release of the anti-inflammatory IL-10 was further increased (1.3-fold change), compared to the B2 treatment alone. This anti-inflammatory effect under simultaneous B2 and EC21a treatment was totally counteracted by the CB2R antagonist SR144528, proving CB2R to be the only target for the EC21a-mediated effect in our experimental settings.

### 2.2. GAT229 Potentiated the CB1R-Mediated Effect of B2 on the Glutamate Release

**Figure 3:**
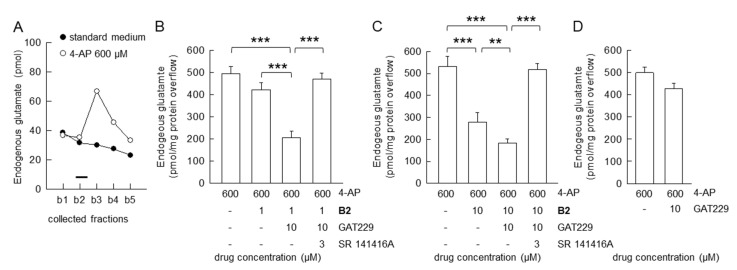
Allosteric modulation of B2: inhibition of the evoked glutamate release from hippocampal nerve terminals in superfusion experiments. (**A**) A representative time-course for the release of endogenous glutamate from nerve terminals is shown. 4-AP was added (2 min; black bar) during superfusion; other experimental details in Materials and Methods. (**B**,**C**) Pharmacological characterization of the novel CB1R agonist B2: inhibition of 4-AP-evoked efflux of endogenous glutamate; antagonism by SR14176A; effectiveness of the CB1R allosteric modulator GAT229 on the B2-mediated inhibition. Bars represent the overflow (pmol/mg protein) of endogenous glutamate in the presence of the drugs at the indicated concentrations. 4-AP was added (2 min) during superfusion: SR141716A, B2 and GAT229 were added starting 8 min before the depolarizing stimulus. (**D**) GAT229 at the used concentrations did not affect the 4-AP-evoked endogenous glutamate release. Data are means ± SD (bars) of n = 3–6 independent experiments performed in duplicate or triplicate. *** *p* < 0.001 and ** *p* < 0.01; one-way ANOVA followed by Bonferroni’s post hoc test.

To evaluate the effects of B2 at the CB1R, and of the positive allosteric ligan GAT229, in the modulation of the B2 activity at the CB1R, we measured the release of endogenous glutamate from superfused purified hippocampal nerve terminals. In superfusion experiments addition of 4-AP, a selective blocker of voltage-activated K^+^ channel able to increase the exocytotic release of neurotransmitters from the nerve terminals evoked a release of the endogenous glutamate, as measured by HPLC (see Figure 3A). We previously reported that both the reference CB1R agonist arachidonyl-2′-chloroethylamide (ACEA) and the compound **B2** inhibited the 4-AP-evoked release of glutamate from the nerve terminals in a way sensitive to the CB1R antagonist SR141716A; antagonism by SR141716A indicated that activation of presynaptic release-inhibitory CB1R was responsible for the effect of ACEA or B2 [21].

We here evaluate the effect of the positive allosteric CB1R modulator GAT229 on the B2 inhibition of the 4-AP-evoked release of glutamate. The compound GAT229, per se ineffective on the 4-AP-evoked release of glutamate, was able to potentiate the effect of B2 at the presynaptic release-inhibitory CB1R. In fact, in the presence of GAT229 (10 μM), with B2 at 1 μM concentration, which was per se ineffective, could inhibit the release of glutamate (Figure 3B). The positive allosteric ligand GAT229 (10 μM; but not 1 μM; data not shown) was also able to increase the inhibitory activity of B2 (10 μM), as shown in Figure 3C. The pharmacological characterization of the receptor involved in the effect of B2 plus GAT229 was completed by performing experiments in the presence of the CB1R antagonist SR141716A. The inhibitory effect of B2, in the presence of GAT229, was totally counteracted by the CB1R antagonist SR141716A (1 μM), proving that the release-inhibitory effect of B2 in the presence of 10 μM GAT229 was accounted for by CB1R activation (Figure 3B,C).

In our experimental conditions, GAT229 did not affect the basal (data not shown) nor on the 4-AP evoked endogenous glutamate release (Figure 3D).

## 3. Discussion

Based on the previously described ability of EC21a to act as allosteric CB2R modulator in a mouse model of neuropathic pain [20], in this paper we investigated its effects on one of the key pathways triggered by CB2R activation, i.e., the regulation of the microglia-mediated neuroinflammation.

In this paper, we report that EC21a strongly potentiates the effect of the CB2R-agonist B2 in an in vitro model of microglia, through the induction of a positive cooperativity and significant modulations of the release of several ILs, thereby mediating an anti-inflammatory activity. Noteworthily, in agreement with the allosteric mechanism, EC21a did not induce any effect in the absence of a CB2R agonist.

Our results show, for the first time, that the combination of CB2R PAM EC21a with the orthosteric agonist B2 significantly counteracts the inflammatory process in microglial cells, through one mechanism that supports the onset, progression and severe symptomatology of different types of diseases. Indeed, the microglial activation and the consequent neuroinflammation play key roles in several neurodegenerative diseases, such as Alzheimer’s disease [22], Parkinson’s disease [23], amyotrophic lateral sclerosis and frontotemporal dementia [24,25], and also, in psychiatric disorders [26]. Evidence highlighted that the balance between promotion and suppression of the inflammatory state is strictly dependent on the phenotype of microglial cells [27,28]. Inflammatory stimuli, such as LPS and interferon (INF-)γ, promote the pro-inflammatory phenotype of microglial cells able to release pro-inflammatory mediators such as IL-1β, IL-6, tumor necrosis factor (TNF-)α, reactive oxygen species (ROS) and NO. In physiological conditions, the resolution of the microglia-mediated inflammatory condition is obtained by a microglial switch from the inflammatory M1 phenotype to the M2 phenotype producing anti-inflammatory cytokines, such as IL-10 [29,30].

To reproduce the inflammatory M1 microglial phenotype, we exposed BV2 cell line to LPS, a commonly used in vitro model of activated microglial cells. As expected, LPS-exposed BV2 cells showed increased release of IL-1β and IL-6, and decreased release of IL-10. The treatment with 1 μM concentration of B2 reverted the IL modulation induced by LPS, reducing the release of both IL-1β and IL-6, and increasing the levels of the anti-inflammatory IL-10.

These data suggest an effective anti-inflammatory action of B2 in microglia cells—mimicking inflammatory conditions by promoting the switch to the neuroprotective M2 phenotype.

The treatment of B2 with a very low concentration of CB2R PAM EC-21a potentiated these effects, halving the release of both IL-1β and IL-6, and increasing the level of the anti-inflammatory IL-10.

These data suggest an effective anti-inflammatory action of this combination in microglia cells through mimicking inflammatory conditions by promoting the switch to the neuroprotective M2 phenotype.

The up-regulation of markers of the M2 phenotype in inflamed microglial cells after activation of CB2R has been already reported after the treatment with several CB2R agonists, i.e., AM1241 [31], JWH133 [32] and β-caryophyllene [33]. Despite the promising potential of CB2R agonists [7,34,35], the main limitations are the common side effects related to internalization of CB2R and off-target activity. Indeed, it has been reported that CB2R agonists can induce internalization and desensitization of the receptor, leading to decreases in signaling and surface receptor levels [36,37,38,39]. As opposed to agonists, PAMs have no intrinsic efficacy and enhance the receptor activation only in the presence of the orthosteric stimulant, allowing better regulation with no or reduced receptor desensitization [40]. Furthermore, one specific signaling profile can further change more or less markedly, when both ligands (orthosteric and allosteric) are bound to the receptor [15,17], and then certain signaling interactions (e.g., G proteins) can be engaged over others (e.g., β-arrestins).

The orthosteric cannabinoid receptor agonist B2 was reported able to activate the CB1R, besides the CB2R [21].

Activation of the CB1R was suggested to provide neuroprotection in MS [41] by limiting the release of glutamate, a key mediator in excitotoxic damage of neuronal cells and oligodendrocytes. In fact, excessive glutamate release and overactivation of glutamate receptors seem to play crucial roles in neuron injury in MS [42,43,44,45]; in other neurodegenerative diseases, such as Alzheimer’s disease, Parkinson’s diseases [45,46,47,48] and amyotrophic lateral sclerosis [49]; and in neuropsychiatric disorders [50]. Reducing the release of glutamate indeed is considered a promising approach in these pathological conditions.

By measuring the endogenous glutamate release from hippocampal glutamatergic nerve terminals, we previously found that these nerve terminals are endowed with release-inhibitory CB1R, while lacking release-modifying CB2R [21]. The compound **B2** was able to inhibit the glutamate release by activating the CB1R, proving to behave as an effective CB1R agonist at presynaptic CB1R, controlling the glutamate release, besides behaving as an agonist at the microglial CB2R.

We can report for the first time that in the presence of the CB1R PAM GAT229 the glutamate release inhibitory effect of B2 was enhanced, and an inhibitory action on the release was revealed at low B2 concentrations, which were per se ineffective at the CB1R. The combination of orthosteric and allosteric ligands at CB1R, therefore, might be used to effectively inhibit the release of glutamate. This combination, by promoting the activation of neuroprotective CB1Rs, might exhibit an effective neuroprotective action in MS, and in other neurodegenerative diseases and neuropsychiatric disorders.

Notably, while inhibition of glutamate release and of excitotoxicity were repeatedly reported to be neuroprotective in MS animal models [42,43,44], combinatorial strategies are likely required to reduce the disease progression and to achieve neuroprotection in patients with secondary progressive MS [42,51].

The compound **B2**, able to activate both CB1R and CB2R, and targeting both the excitotoxic injury and neuroinflammation, might indeed offer a useful approach to treat MS and neurodegenerative disorders in which the excitotoxic cascade and neuroinflammation are activated.

It is to be noted that the CB1R PAM GAT229 made B2 capable to activate the CB1R, in addition to the CB2R, at low concentrations. Indeed, B2 concentrations exhibiting effective anti-inflammatory action and promoting the microglial switch to the neuroprotective phenotype, but unable to control the glutamate release, were proven effective on glutamate release in the presence of GAT229. As far as the CB1R is concerned, although the receptor seems to also be involved in maintaining the glutamate NMDAR activity within safe limits, thereby protecting neural cells from excitotoxicity; although the direct activation of the receptor seems responsible for effects of other long-time known drugs such as the analgesic paracetamol; and although targeting the CB1R is considered a proper direction against neurodegenerative diseases and neuroinflammation [52]—the possible psychoactive effects of the receptor activation via orthosteric ligands cannot be ignored.

The negative side effects associated with CB1R activation mainly occur when the receptor is activated at supraphysiological levels, disrupting normally tightly controlled spatial and temporal activation. Use of positive allosteric modulators may allow a pharmacological fine-tuning of activation, enhancing receptor activity, and at the same time maintaining spatial and temporal control, resulting in an improved safety profile over the direct orthosteric activation.

On these bases, the co-administration of a CB1R orthosteric agonist with a positive allosteric modulator of CB1R could determine a lack of side effects, maintaining the beneficial effect due to a CB1R direct activation [53,54,55].

Finally, another strategy to attenuate neurotoxicity and neuroinflammation is represented by the indirect activation of CBRs via MAGL or FAAH inhibition [52]. However, blocking the activity of these enzymes is not devoid of side effects due to an increase in the availability of endocannabinoids [56,57]. In this context, the effects of combining an allosteric positive modulator such as EC-21a or GAT229 with an inhibitor for FAAH or MAGL also deserve to be investigated for a comprehensive approach to the treatment of neurodegenerative diseases.

In summary, our results support the hypothesis that the combination of a dual orthosteric agonist of CBRs with positive allosteric modulators of CBRs could represent a promising therapeutic approach to the treatment of neurodegenerative disorders.

## 4. Materials and Methods

### 4.1. Synthesis of N-[5-bromo-1,2-dihydro-1-(4′-fluorobenzyl)-4-methyl-2-oxo-pyridin-3yl]Cycloheptanecarboxamide (EC21a)

**Scheme 1:**
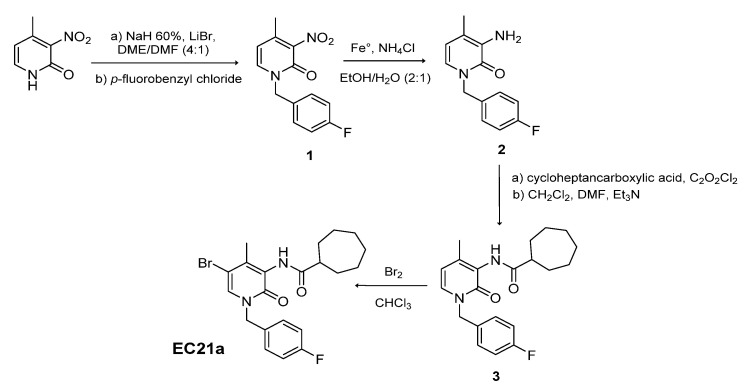
Synthesis of *N*-[5-bromo-1,2-dihydro-1-(4′-fluorobenzyl)-4-methyl-2-oxo-pyridin-3yl] cycloheptanecarboxamide (EC21a).

This compound was obtained modifying the procedure previously reported [20].As described in Scheme 1, 2-hydroxy-4-methyl-3-nitropyridine was N-alkylated by treatment with *p*-fluorobenzyl chloride in anhydrous *N,N*-dimethylformamide (DMF) and 1,2-dimethoxyethane (DME) in the presence of NaH and LiBr at 65 °C for 24 h, yielding compound **1**. Compound **1** was then reduced to the corresponding amine derivative 2 by refluxing at 80 °C for 3 h with iron powder, ammonium chloride in ethanol/H_2_O (ratio of 2:1). The reaction between the amine derivatives 2 and the cycloheptanecarbonyl chloride in DMF and triethylamine initially at 0 °C and then at room temperature for 12 h, gave the corresponding amide 3. Finally, the desired 5-bromo derivative EC21a was obtained by bromination of compound **3** in chloroform with a solution of Br_2_ in CHCl_3_ at 0 °C and then at room temperature for 12 h. EC21a was purified by flash chromatography on silica gel using petroleum ether/ethyl acetate 7:3 as eluent. Yield: 66%.

### 4.2. Synthesis of N-Cycloheptyl-1,2-dihydro-5-bromo-1-(4-fluorobenzyl)-6-methyl-2-oxo-pyridine-3-carboxamide (***B2***)

**Scheme 2:**
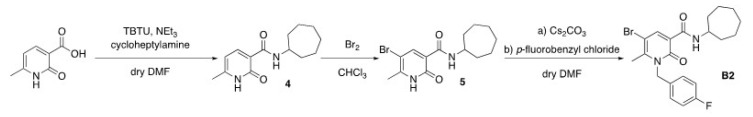
Synthesis of *N*-cycloheptyl-1,2-dihydro-5-bromo-1-(4-fluorobenzyl)-6-methyl-2-oxo-pyridine-3-carboxamide (**B2**).

Compound **B2** was prepared following the synthesis reported in Scheme 2. The commercially available 2-hydroxy-6-methyl-nicotinic acid was treated with the coupling agent 2-[(1H-benzotriazole-1-yl)-1,1,3,3-tetramethyluronium tetrafluoroborate] (TBTU) in the presence of triethylamine at 0 °C for 30 min. After that, the cycloheptylamine was added and the reaction mixture was stirred at 0 °C for 30 min and then at room temperature for 12 h to give the carboxamide derivative 4. The treatment of intermediate 4 with a solution of bromine in chloroform afforded the corresponding bromo-derivative 5, which was N-alkylated by treating with cesium carbonate in anhydrous DMF at room temperature for 1 h, and then with *p*-fluorobenzyl chloride, to give the desired compound **B2** [21].

### 4.3. Cell Line and Reagents

The BV-2 murine microglial cell line is an immortalized cell line with morphological, phenotypic and functional properties associated with freshly isolated microglia, and thus, it is frequently used as in vitro model to study microglial responses to pharmacological stimuli [58,59]. The BV-2 cells were cultured in high glucose Dulbecco’s modified eagle’s medium (Corning, Tewksbury, MA, USA) supplemented with 10% fetal bovine serum (FBS), streptomycin (100 g/mL) and penicillin (100 units/mL) (Sigma-Aldrich, Milan, Italy).

Lipopolysaccharide (LPS) (Escherichia coli 0111:B4) was purchased from Sigma-Aldrich (Milan, Italy) and SR144528 was from Tocris (Bristol, UK).

These reagents were stocked in dimethyl sulfoxide (DMSO) at 4 °C and diluted to the final concentration into cell culture medium just prior to the experiments. The DMSO concentration was always maintained <0.1% in order to have no cytotoxicity and no significant effect on the specific assays.

GAT229 was purchased from Sigma-Aldrich (Milan, Italy); 4-aminopyridine (4-AP), arachidonyl-2′-chloroethylamide hydrate (ACEA) was from Tocris (Bristol, UK); SR 141416A was from Selleckchem (Munich, Germany); 4-AP was dissolved in physiological medium. Stock solutions of the drugs were prepared in dimethylsulfoxide and diluted at least 1:1000 in physiological medium; stock ACEA solution was 13.7 mM in oil and then diluted to the final concentration in physiological medium; dimethylsulfoxide diluted 1:1000 had no effect on endogenous glutamate release.

### 4.4. Analysis of Interleukin Release after LPS Stimulus

The concentrations of the pro-inflammatory IL-1β and IL-6, and the anti-inflammatory IL-10, were determined by specific ELISA assays (MyBioSource, San Diego, CA, USA) on the collected culture media.

Microglial cells were treated with the orthosteric agonist, B2 (1 μM), in the presence or absence of the CB2R positive allosteric modulator, EC21a (0.1 μM), for 30 min and then stimulated with LPS (5 μg/mL) for 4 h. When the CB2R antagonist (SR144528, 1 μM) was administered, it was added 15 min before agonist. All treatments and the LPS stimulation were performed in FBS-free medium, because serum showed to interfere with interleukin release (data not shown) and several commercial FBS contain endocannabinoids that could influence cellular experiments [60].

### 4.5. Assessment of Glutamate Release

Adult rats (males, Sprague Dawley, 200–250 g) were housed at the animal care facility of the Department of Pharmacy (DIFAR), University of Genova, Italy, with constant parameters (temperature: 22 ± 1 °C; relative humidity: 50%; and with 12 h light on/off cycles). The animals had food and water ad libitum. The animals were used following the principles and procedures outlined in the EU guidelines (2010/63/EU) and the Italian Legislative Decree number 26/2014, and were approved for use by the Italian Ministry of Health (protocol number 30/11/2016-OPBA of November 2016), in accordance with the Ministerial Decree 116/1992. The researchers made all efforts to reduce the number of animals used and their suffering.

Briefly, the hippocampus was removed and placed in ice-cold 0.32 M sucrose, buffered at pH 7.4 with Tris-HCl. The purified nerve terminals (synaptosomes) were prepared as reported previously [21,61,62]. The hippocampus was homogenized in sucrose; the homogenate was centrifuged (1000× *g* for 5 min at 4 °C), and the discontinuous Percoll gradient (2, 6, 10 and 20% (*v*/*v*) in Tris-buffered sucrose) procedure was used to isolate synaptosomes from the interface between 10 and 20% Percoll. The synaptosomes were then washed in standard HEPES medium by centrifugation. The freshly isolated synaptosomes were not contaminated by astrocyte processes, microglia or oligodendrocytes [63,64,65,66].

Notably, the release of neurotransmitters can be measured from synaptosomes in superfusion, allowing the pharmacological characterization of compounds directly acting at the presynaptic release-regulating receptor target [21,61,62,67]. The release of glutamate was measured from hippocampal synaptosomes in superfusion [21]. During superfusion, 3-min samples (from b1 to b5) were collected starting from *t* = 33 min until the end of the experiment. At *t* = 38 min the synaptosomes were superfused with standard medium added with the depolarizing compound 4-aminopyridine (4-AP, 600 μM). To evoke the release of glutamate, we added in the superfusion medium the depolarizing compound **4-AP**, a selective blocker of voltage-activated K^+^ channel which has been repeatedly reported to be able to increase the exocytotic release of glutamate from synaptosomes, in a way sensitive to modulation by presynaptic release-modulatory receptors [68,69,70]. The CB1R agonist (B2 1 μM), CB1R antagonist (SR 141416A, 1 μM) and CB1 allosteric compound (GAT229 1 μM or 10 μM) were added (in respective tests) 8 min before the depolarizing stimulus and maintained in the presence of 4-AP. The CB1R agonist or antagonist or allosteric compound had no effects on the basal release of glutamate. As a control, in each superfusion experiment, at least 2 chambers were superfused only with the standard medium. The glutamate released in the collected samples was measured by HPLC analysis (Waters Alliance, Milford, MA, USA) involving automatic pre-column derivatization with *o*-phthalaldehyde, separation on a C_18_ reverse phase chromatography column (Chrompack International, Middleburg, The Netherlands) and fluorimetric detection. Homoserine was used as the internal standard; the detection limit was 100 fmol/μL. [71,72]. In Figure 2A a representative time course of endogenous glutamate released in the collected samples in a representative experiment is shown. The amounts of endogenous glutamate present in the collected samples were expressed as pmol/mg protein.

The effect of the compounds (or depolarization) on the glutamate efflux was calculated as overflow by subtracting the basal release in the control chambers from the total amount of glutamate released during stimulation in substance-treated chambers (or in chambers supplemented with 4-AP).

### 4.6. Statistical Analysis

The results were shown as mean ± standard deviation (SD) of at least three independent experiments. Statistical analysis was performed by commercial software (GraphPad Prism, San Diego, CA, USA) using ordinary one-way ANOVA followed by Tukey’s multiple comparison test. A *p*-value < 0.05 was considered statistically significant.

## Abbreviations

ECS	Endocannabinoid system
AEA	Anandamide
2-AG	2-arachidonoylglycerol
FAAH	Fatty acid amide hydrolase
MAGL	Monoacylglycerol lipase
CB1R	Cannabinoid receptor type 1
CB2R	Cannabinoid receptor type 2
PAMs	Positive allosteric modulators
GAT229	R-(+)-3-(2-Nitro-1-phenylethyl)-2-phenyl-1H-indole
B2	*N*-cycloheptyl-1,2-dihydro-5-bromo-1-(4-fluorobenzyl)-6-methyl-2-oxo-pyridine-3-carboxamide
EC21a	*N*-[5-bromo-1,2-dihydro-1-(4′-fluorobenzyl) -4-methyl-2-oxo-pyridin-3yl]cycloheptanecarboxamide
